# Understanding the medication experience of patients with advanced non-small cell lung cancer taking epidermal growth factor receptor-tyrosine kinase inhibitors: A phenomenological study

**DOI:** 10.1371/journal.pone.0286333

**Published:** 2023-05-30

**Authors:** Meng-Jung Wen, Han-Lin Hsu, Chia-Lun Chang, Jou-Han Wang, Chun-Nan Kuo, Yen-Chun Hsin, Elizabeth H. Chang

**Affiliations:** 1 Department of Clinical Pharmacy, School of Pharmacy, College of Pharmacy, Taipei Medical University, Taipei, Taiwan; 2 Division of Pulmonary Medicine, Department of Internal Medicine, Wan Fang Hospital, Taipei Medical University, Taipei, Taiwan; 3 School of Respiratory Therapy, College of Medicine, Taipei Medical University, Taipei, Taiwan; 4 Division of Hematology and Medical Oncology, Department of Internal Medicine, Wan Fang Hospital, Taipei Medical University, Taipei, Taiwan; 5 Department of Internal Medicine, School of Medicine, College of Medicine, Taipei Medical University, Taipei, Taiwan; 6 School of Public Health, College of Public Health, Taipei Medical University, Taipei, Taiwan; 7 Department of Pharmacy, Wan Fang Hospital, Taipei Medical University, Taipei, Taiwan; 8 Research Center for Pharmacoeconomics, College of Pharmacy, Taipei Medical University, Taipei, Taiwan; The Hong Kong Polytechnic University, HONG KONG

## Abstract

**Background and objective:**

Patients with cancer taking oral antineoplastic medications may encounter problems including suboptimal adherence as well as physical and psychological disease burden. Despite increase in the use of oncology pharmacy services, there are wide variations between healthcare professionals and patient perceptions of patients’ medication experiences. The objective of the study was to explore the medication experience of taking oral targeted therapy in patients with advanced non-small cell lung cancer (NSCLC).

**Method:**

We purposively sampled advanced stage (stage III or IV) NSCLC patients taking epidermal growth factor receptor-tyrosine kinase inhibitors (EGFR-TKIs) in a medical center in Taiwan. Face-to-face interviews using semi-structured interview guides were conducted. Interviews were transcribed verbatim and thematic analysis was applied. A phenomenological methodology was adopted to explore the underlying meaning of patients’ lived experience.

**Results:**

A total of 19 participants with a mean age of 68.2 years were interviewed. The duration of EGFR-TKIs use ranged from 2 weeks to 5 years. When first learned about the unexpected yet ‘treatable’ cancer, participants expressed strong emotional responses based on their intrinsic beliefs of the terminal disease and therapy. They walked along an unfamiliar trail while confronting physical and psychological challenges and made compromises to treatment. Gaining experiences from cancer journey, patients with cancer continuously seek the ultimate goals–‘return to normal’.

**Conclusions:**

This study also revealed medication experiences of participants’ journey from seeking information in the initial phase and living with cancer, to taking back control of their own lives. Healthcare professionals could better empathize with patients’ loss of control and understand their perspectives when making clinical decisions. These findings can guide interdisciplinary teams to integrate patients’ beliefs and conduct pre-screening assessments of health literacy levels to tailor communication. Subsequent interventions should be developed to identify barriers to medication self-management and empower patients by building social networks.

## 1. Introduction

Lung cancer is the leading cause of cancer deaths and is associated with rising costs of therapy in recent years [1, 2]. First-line treatment for advanced or metastatic non-small cell lung cancer (NSCLC) is chosen based on the presence of gene mutations [3]. Oral targeted therapy, specifically with epidermal growth factor receptor-tyrosine kinase inhibitors (EGFR-TKIs), is listed as initial therapy, especially for patients in East Asia. Detection rates of EGFR mutations are over twice as high among Asians as it is for people in Western countries, [4] which warrants the exploration of medication use experiences in this patient population.

Compared to intravenous cancer therapies, oral cancer medications are favored because of their convenience; however, suboptimal medication adherence may significantly undermine the effectiveness of such medications [5]. Roughly one-fifth of cancer patients fail to achieve adequate adherence, presenting under- or over-adherence after long-term use [6, 7]. Non-adherence to oral cancer medications can lead to early disease progression, more hospitalizations, higher mortality, and higher healthcare expenditures [8, 9]. As such, factors associated with low adherence in terms of patient perceptions, such as medication beliefs or preferences and self-management needs, should be further explored [10].

Patient’s experiences with medication use across different chronic diseases has been widely investigated and could be utilized to identify drug therapy problems and medication-related issues. Medication experience is defined by Shoemaker et al., is a practical concept to describe an individual’s holistic experience related to medication use [11]. A 2016 meta-synthesis focusing on the exploration of negative experiences in medication use and manifested the need of reducing medication-related burden from medication-taking practice [12]. As such, by utilizing medication experience in patients’ daily live, healthcare professionals can effectively identify unnecessary treatment and prevent adverse drug reactions.

Different from patients with other chronic diseases, advanced cancer patients often perceive both high necessity and great concern about their medication [13]. These ambivalent beliefs about their medication, derived from prior experiences and from physical and psychological factors, may change over time due to treatment-related events [14]. Although associations between medication beliefs and other treatment factors were established, few studies have qualitatively investigated the formation of positive or negative beliefs or how they moderate medication-taking behaviors during the treatment trajectory [14]. Moreover, unlike traditional intravenous treatments which can be closely monitored by healthcare professionals (HCPs), self-management, including disease management, lifestyle modifications, and help-seeking, is an inevitable part of patients using oral cancer medications [15]. Thus, with more than half of patients being diagnosed at over 65 years of age, patients with advanced cancer encounter many challenges with medication-related self-management and struggle to achieve optimal adherence [16, 17].

Previous studies of patients’ perceptions of oral cancer medications and corresponding interventions were conducted in patients with chronic myeloid leukemia and breast cancer, which generally have longer survival periods in more-stable conditions [18, 19]. However, due to differences in cancer trajectories in patients with NSCLC using EGFR-TKIs, understanding patient perceptions regarding oral cancer medications can help tailor evidence-based practice with increasing oncology care. In addition, psychosocial burden experienced by patients with cancer was resulted from cancer-related stigmatization, cultural backgrounds could thus impact on patients’ subjective experiences of taking medication [20]. However, most existing studies were limited in Western countries [20–22], patients in East Asia encounter different cultural perceptions of medication use structural factor of oncology health care services. Therefore, the purposes of this study were to describe and interpret the medication experiences and meaning of taking EGFR-TKIs for patients with NSCLC.

## 2. Materials and methods

### 2.1. Design

This qualitative research adopted a hermeneutic phenomenological philosophy which can give insights into the essence of cancer patients’ medication experiences as well as the meaning of taking EGFR-TKIs in their daily lives [23]. The meaning of medication-taking behaviors was obtained by exploring patients’ lifeworld and the interpretation of their medication experience in the context of cancer journey [23]. This study was conducted from May 2019 to January 2020.

### 2.2. Participants and settings

After the study was approved by the Taipei Medical University-Joint Institutional Review Board (TMU-JIRB, No. N201712063), purposive sampling was used to recruit participant with varied characteristics including age, cancer stage, treatment duration, and TKI type in a medical center in Taiwan. Eligibility criteria included participants (1) aged over 20 years, (2) with a diagnosis of NSCLC, (3) who were receiving treatment with EGFR-TKIs, and (4) could communicate and participate in interviews in Mandarin Chinese or Taiwanese for about 30~60 min. Exclusion criteria were those who could not express their opinions.

Participants were recruited from two outpatient clinics (pulmonology and oncology) in a medical center. Two physicians approached potential participants who met the inclusion criteria and briefly introduced the study information during their visit. If patients were interested in the study, they were referred to the research team members outside of the clinic to further explain the details of the study. The potential participants were allowed to review the study information for 10–15 minutes and raise any questions. The research team members also respect the willingness to participate. The written informed consent process was conducted and obtained from all participants before conducting the interview. During this process, researchers thoroughly explained the details including the purpose, format, and length of the interview. We also assured participants of confidentiality issues and the freedom to withdraw from the study.

### 2.3. Data collection

Individual face-to-face, in-depth interviews were conducted in a private room in one medical center. A semi-structured interview guide derived from the medication experience framework is shown in Table 1 [11]. The first draft of the interview guide was revised by a qualitative researcher and opinions from pilot testing. Before each interview, a questionnaire was administered to collect participants’ demographic information. A questionnaire was designed to collect participants’ demographic information before the interview. Before conducting each interview, a reflexive process was conducted by the interviewer of writing down propositions, assumptions, and influences which demonstrated an awareness of the researcher’s limitations and plans to minimize bias. All interviews were audio recorded and the interview data was stored in an encrypted device.

**Table 1:** Semi-structured interview guide.

Domains	Interview Questions
A meaningful encounter	1. What does it feel like to be diagnosed with non-small cell lung cancer? 2. What did it feel like when you began treatment? 3. What does ’medication-taking behavior’ mean to you? 4. What do you think about this medication (targeted therapy)?
Bodily effects	5. What bodily effects do taking this medication cause you to feel?
Unremitting nature	6. What does it feel like when you need to take this medication for a long time? 7. What problems/worries have you had during your treatment?
Exert control	8. How did taking this medication change your daily life? 9. What are your coping strategies?

### 2.4. Data analysis

Demographic questionnaires were analyzed using descriptive statistics to describe participant characteristics. All interviews including two plot testing were transcribed verbatim, and all personal information was de-identified. Qualitative data was analyzed by the first author who has intensive methodological training in her graduate study. Atlas.ti version 8.0 (Scientific Software Development, Berlin, Germany) was employed to analyze all transcript and manage data. Theme analysis guided by van Manen was used to explore the essence of cancer patients’ medication experiences in their daily lives through four existential elements, including spatiality (lived space), corporeality (lived body), temporality (lived time), and relationality (lived relationships) [23]. Three processes of theme analysis were used to discover and create meaning of participants’ lived experience description. In the first process—holistic reading approach, all interview transcripts were read and reread to grasp the overall meaning of the study phenomenon by continuously asking the main research question: ‘What do patients with NSCLC describe and interpret their medication experience of taking EGFR-TKIs?’ Researcher then reflected each experiential text and identified with common features and structure as essential themes by utilizing the selective reading approach. The essential themes were coded by some vivid expressions or phrases used by participants or borrowed from their everyday life. The detailed reading approach was used to investigate every single sentence to search for answers of ‘what may this sentence reveal the medication experience of this participant?’ Lastly, both essential and unique essential themes were concluded into a higher level that overarching the whole structure of medication experiences to present the phenomenon [23].

### 2.5. Rigor

The rigor of this qualitative research was established based on Lincoln and Guba’s four principles [24]. Researchers immersed themselves into interview data to gain a general understanding of patients’ experiences first and read line-by-line to capture the meaningful details of patients’ perceptions to ensure prolonged engagement. Qualitative transcript was coded and analyzed by two independent researchers to ensure the consistency among codes and themes to establish credibility. Peer debriefing with members in cancer advisory broad was regularly conducted to ensure the interpretations and emergent themes were directly derived from data. Thick description of procedure, data collection and analysis were provided to confirm the findings can transfer to other settings. To enhance confirmability, data management using qualitative software to keep all coding trails. As well, memo writing was utilized to keep records on any changes of codes and themes. Research processes were detailed described and use the Consolidated Criteria for Reporting Qualitative Studies (COREQ-32) checklist to assess the study quality in S1 Checklist [25].

## 3. Results

In total, 19 participants consisting of five men and 14 women, with a mean age of 68.2 years, were approached and recruited in a medical center in Taiwan. Participant demographics varied and are shown in Table 2. According to the self-reported health condition, participants were in an advanced stage (stage III or IV) of NSCLC. The duration of taking TKIs ranged from 1 month to 5 years, with about half of the participants at less than 1 year and others were above 1 year. Four types of TKIs taken by the participants were covered all indicated EGFR-TKIs in Taiwan. The range of interview lasted from 30 to 60 minutes. In Table 3, four existential themes were identified with subthemes and representative quotes, and provided an overall picture of NSCLC patients using EGFR-TKIs in their daily lives. Our data matrix of complete sets of themes, subthemes and corresponding codes is listed in S1 Data.

**Table 2:** Characteristics of study participants.

Characteristic (*N* = 19)	No. of participants (%)
Female	14 (73.7)
Age in years (mean, SD)	68.2, 13.3
Educational attainment	
Elementary school	7 (36.8)
Junior high school	1 (5.3)
Senior high school	8 (42.1)
College	1 (5.3)
Graduate school	2 (10.5)
Marital status	
Married	19 (100)
Employment status	
Working	2 (10.5)
Homemaker	7 (36.8)
Retired	10 (52.6)
Living arrangement	
With spouse and children	9 (47.4)
With spouse	4 (21.1)
With children	6 (31.6)
Stage at diagnosis	
III/IV	19 (100)
Chemotherapy	
Had undergone chemotherapy	5 (26.3)
Chemotherapy naïve	14 (73.7)
Current type of tyrosine kinase inhibitor	
Gefitinib	10 (52.6)
Erlotinib	3 (15.8)
Afatinib	5 (26.3)
Osimertinib	1 (5.3)
Duration of therapy	
<3 months	2 (10.5)
<1 year	7 (36.8)
>1 year	10 (52.6)
Complementary alternative medicine use	
Traditional Chinese medicine	8 (42.1)
Nutritional supplements	9 (47.4)

**Table 3:** Themes, subthemes and representative quotes of medication experience of patients with non-small cell lung cancer using oral targeted therapy.

Themes	Subthemes	Representative quotes
Temporality: Values and expectations of oral targeted therapy in the terminal sentence	Unexpected death sentence from a ‘treatable’ disease	*“The doctor suggests treat (the targeted therapy) as cardiovascular medicine that I have taken for a long time*, *and it could control (cancer)*. *It is actually whimsical*…*The tumors will pop out at any time and is still a bit dangerous*.*” TKI-01*
		*“I’m lucky because (the medication) was covered by the National Health Insurance*… *I was really happy that I’m able to take the medicine (EGFR-TKIs) and can first take oral medicine*, *rather than undergoing chemotherapy*. *Then*, *however*, *I regretted taking the medicine after having skin ulcers all over my face*.*” TKI-06*
	Struggling to stay motivated about taking medications over the long journey	*“How do you make sure your cancer is completely gone by taking this medicine*? *Is that possible*? *Whether the medicine works for better or worse*, *you have to take it*. *It’s hard to say what’s going to happen after taking it (the medicine)*.*” TKI-11*
Corporeality: Living with adversities	Managing disease-related issues	*“It was really shocking*, *and I kept crying*. *Life is so hard… and I kept questioning the reasons why this (the cancer diagnosis) happened to me*. *I actually didn’t want to face it (the cancer diagnosis and the treatment) at that time*. *Also*, *I didn’t ask the doctor any questions*, *including the stage of my cancer*.*” TKI-06*
		*“Although I watched some health-related series on TV*, *the information was not very complete…*. *I also got messages from other friends*, *but I think it (that information) didn’t help much*.*” TKI-15*
	Distress with medication side effects	*“The dermatologist told me that this is a side effect of taking the medicine*. *It will do no good to apply anything*. *I was so frustrated at that time*. *I couldn’t move my hands*.*” TKI-02*
Relationality: Cancer diagnosis as a stepping stone or stumbling block in interpersonal relationships	Reorienting relationships	*“My relationship with my father is actually considered to be very traditional Chinese father-and-son*. *When we went on a family trip*, *I was shocked and embarrassed because my dad suddenly held my hand*.*” TKI-16*
		*“My scalp condition (pimples) was caused by medications (not contagious)*, *but he (the hairstylist) refused to cut my hair*.*” TKI-10*
	Communication and conflicts about medication issues	*“[After receiving bad lab reports of cancer markers*,*] I didn’t dare tell the doctor that it was because of taking Chinese medicine*, *or if it was because [Drug A] didn’t work so well*.*” TKI-17*
Spatiality: Changing lenses to bounce back to normal	Searching back and forth for an acceptable answer	*“I applied the prescribed ointment for a week*, *but they [my sores] became more and more serious*. *Hence*, *I applied Mentholatum (an over-the-counter medicine) later*, *and my face improved*. *So*, *I think it [Mentholatum] fits me just fine*.*” TKI-17*
		*“My friends even said that I should treat this (cancer) as a chronic disease (laughs)*. *…As if I’m taking medicine for lowering blood pressure*, *which I need to take every day*.*” TKI-02*
	Leveraging social support to keep the faith	*“What you have to do is to appease them (new patients) first*. *…I am a person who went through this (process)*, *so I will counsel them based on my own experience*. *I think they’ll be much better (after hearing my words)*.*” TKI-16*
	Back in the driver’s seat	*“My body will tell me what I am supposed to do*. *For example*, *I’ll always bring my sunglasses*. *If my eyes feel uncomfortable*, *wearing them can protect my eyes*.*” TKI-02*
		*“People shouldn’t encourage the patient too much*. *Likewise*, *don’t remind them that they have cancer or tell them what to do; just encourage them to live like a normal person*.*” TKI-07*

### 3.1. Temporality: Values and expectations of oral targeted therapy in the terminal sentence

#### 3.1.1 Unexpected death sentence from a ‘treatable’ disease

Although they had been given the alternative to be treated with oral targeted therapy, participants still viewed the cancer diagnosis as a sudden and momentous occurrence. Pessimistic views of having a cancer diagnosis originated from prior experiences in most cases. All participants expressed a huge shock as well as feelings of grief when they linked their diagnosis with similar situations they had heard about from other cases. Participants repeatedly mentioned having ‘terminal stage’ cancer as if it were ingrained in their living or lifestyle choices.

The meaning to each participant of the medication given varied due to different personal experiences. Notedly, almost all patients’ perception of diseases interacted profoundly with their perception about targeted therapy when they reflected their experiences of knowing their cancer diagnosis. Although some may have felt that being able to take oral targeted therapy shed a light on their life-threatening disease, the condition was not always as good as they expected.

#### 3.1.2. Struggling to stay motivated about taking medications over the long journey

Participants continually evaluated whether it was worthwhile continuing to take their medication in the long-term by weighing potential risks and benefits. On the one hand, some participants held positive views through objective assessment, such as imaging, laboratory values, and comparison of other cases to remain on the medication. On the other hand, although participants knew of the unknown effectiveness, recurrence, or undesirable adverse effects, they still passively accepted the medicine because they had no other choices.

### 3.2. Corporeality: Living with adversities

#### 3.2.1. Managing disease-related issues

During their cancer journey, almost all participants experienced suffering not merely from physical pain but also from extreme psychological hardship. When encountering enormous cancer-related challenges in daily life, participants were eager to find the best solution by trying a variety of approaches. Among the challenges, they specifically desperately wondered about the reasons for having lung cancer.

Almost all participants responded that they began to search for useful advice after the diagnosis. Apart from their attending physician, participants generally mentioned searching various sources of information from media, friends, family members, and other HCPs.

Instrumental support is also needed when managing oral EGFR-TKIs. Several participants illustrated how they utilized personal tips to ensure that they took their medication as prescribed. For example, they used a pillbox that they loaded themselves or by family members to properly manage concomitant medications. Moreover, connecting the habit of taking medicine with everyday tasks helped them take their medication once they saw something in their daily life as a reminder.

#### 3.2.2. Distress with medication side effects

Participants reported many difficulties when managing oral EGFR-TKIs. During the treatment trajectory, participants often suffered multidimensional distress when common adverse effects, such as skin rashes, diarrhea, and paronychia occurred.

Also, participants had to cope with financial stress during treatment. Several participants viewed the heavy medical expenses as one of the ‘side effects’ that forced them to encounter early retirement or a period of sick leave, or to feel despair over paying the bill of out-of-pocket medicine. These treatment-related events, embedded in long-term stress from the cancer diagnosis, placed a large burden on these patients with cancer.

### 3.3. Relationality: Cancer diagnosis as a stepping stone or stumbling block in interpersonal relationships

#### 3.3.1. Reorienting relationships

The cancer diagnosis was a spotlight that lit up participants and dramatically changed their relationships with other people. Having lung cancer meant living with a life-threatening condition, and participants noted that they suddenly attracted much attention from friends and family members. In most cases, a cancer diagnosis was a strong stimulus for a family to connect more strongly. With wholehearted support, participants typically described how they cherished time with their family by seriously dealing with cancer. However, other participants revealed that rapid changes in interpersonal relationships had inversely worsened their social connections.

Participants also discussed a sense of alienation after their diagnosis, as they were unwilling to become a heavy burden on their family. For example, one elderly woman admitted that for the sake of relieving her children’s stress, she pretended that she did not know about her health condition, since her children had never told her the truth about having lung cancer.

Furthermore, participants stated that living with cancer somewhat disturbed their previous daily routines, and they maintained a distance from strangers. Another social disturbance even led to the stigma attached to changes in their physical appearance caused by medication side effects. For example, one woman with drug-induced acne on the head reported how strangers misunderstood that her acne was contagious.

#### 3.3.2. Communication and conflicts about medication issues

Although most participants were highly satisfied with their oncologists and discussed disease-related issues with them, a lack of two-way communication still occurred in several participants’ experiences. Participants mostly felt that they had no problems with their doctors and trusted those professionals, possibly due to the hierarchical nature of patient-physician relationships. However, conflicts of perception still occurred between patients and HCPs resulting from inadequate information exchange. For instance, several participants revealed that they did not fully disclose their discomforting symptoms or complementary alternative medicine (CAM) use to HCPs. Participants further explained the underlying reasons for hiding their true feelings during medical visits: that they were afraid of causing a disturbance or of having a negative experience.

The preferred communication style as to the extent of revealing the facts about their health condition varied among participants. Some felt they had the right to receive all of the information whether it was good or bad news, while others were unwilling to know every detail about their treatment. One woman stated that the information from HCPs failed to meet her actual needs. She preferred to receive explicit and achievable instructions to manage her disease rather than statistical data on the time of survival. Some of the participants expect their healthcare professionals to have more empathy about their side effects resulting from the medicine they were taking.

### 3.4. Spatiality: Changing lenses to bounce back to normal

#### 3.4.1. Searching back and forth for an acceptable answer

After experiencing numerous trial-and-error processes in trying to cope with cancer-related challenges, participants gradually downgraded their initial expectations and identified tolerable approaches that best suited them. Almost all participants recognized that complex medical information was hard to manage, and they were thus forced to seek help. Apart from the attending physician, seeking medical advice from a second opinion or an HCP in another specialty for assurance was acceptable in some participants’ cases. Furthermore, participants often needed to deal with a series of failures in managing side effects in order to achieve a balance.

Emotional distress is another major problem when living with cancer, which forced participants to gradually develop their own coping strategies. Facing difficulties that may never have solutions, participants underwent a transformation by making compromises in the face of this reality. For example, several participants indicated changes to previous habits that they attributed to their having cancer. Also, some participants kept faith in the treatment effectiveness by gaining hope for a new drug, other successful cases, or changes in perspectives toward targeted therapy.

#### 3.4.2. Leveraging social support to keep the faith

Most participants demonstrated the determining factor for them hanging in there was whole-hearted support from their family and friends. At the beginning, participants experienced changes in the nature of their interpersonal relationships; although as time went by, they had to keep moving forward by themselves in the face of adversities. Different expectations of peer support groups were held by some participants. Participants who joined peer activities reported that they were highly satisfied with the available resources. Both information and emotional connections were reported when utilizing peer support online resources. Information such as updated treatment trends, ongoing clinical trials, and the cost of out-of-pocket medications was useful for newly diagnosed patients. They also appreciated having a feeling of belonging by sharing the same predicament. However, participants with a longer treatment duration reported that they might not have further needs and were willing to change roles from receiving to giving.

#### 3.4.3. Back in the driver’s seat

By changing from passive acceptance to active commitment to treatment, participants broke the deadlock on previous arguments and took back control of their life. For example, some participants who underwent a long period of treatment felt they had moved to a space where they could breathe more easily due to taking a treatment holiday or modifying their routine to take medication. Also, participants even described that they increasingly got used to living with cancer and had accommodated to external changes.

Furthermore, breaking cancer stereotypes by putting the propositions aside helped participants stay independent and return to a ‘new’ normal. For example, several participants suggested people should avoid paying excessive attention to cancer patients at this stage.

## 4. Discussion

Medication experiences from participants’ perspectives in patients with NSCLC using oral targeted therapy were highly interrelated with their daily lives. These holistic views of participants encompass the physical and psychological effects and social disturbances of taking EGFR-TKIs. Although views on cancer and targeted therapy were dictated by previous experiences, participants developed their own coping strategies after encountering disease-related challenges and relationship transitions. Most participants continued to take EGFR-TKIs as they had no other choices to save their life, but they still tried their best to accommodate the situation by changing their mindset toward the disease and the treatment.

In this study, patients with metastatic NSCLC seemed to differentiate their disease from other chronic conditions. Similar to Thorne et al.’s themes, patients had negative impressions of the disease and anxiety over having an incurable disease, typically based on hearing about other people’s cases [26]. Although they gradually gained personal experiences of living with cancer, the threat of death due to having advanced lung cancer could not be overlooked due to uncertainties about cancer recurrence or metastasis. Such views could have major implications for how healthcare professionals communicate with their patients. For example, the analogy of taking chronic oral medications for NSCLC versus high blood pressure medications might not be convincing for patients without appropriate explanations. This finding also sheds the light on integrating patients’ beliefs or other psychosocial factors into shared decision-making to promote patient-centeredness and address communication discordance.

Behavioral coping strategies for medication-related issues in the initial phase of treatment were widely reported, while individualized information for managing one’s daily routines is lacking. To deal with medication-related issues, participants in this study developed several self-management abilities using personal reminders, modifying regimens, or adopting CAM. Similar findings demonstrated that appraisals of different methods may vary, but all patients experienced several trial-and-error processes in learning to manage their problems [27]. Simultaneously, extreme emotional distress from cancer’s notorious reputation and the unclear benefits of oral antineoplastic agents can also lead to early searching behaviors [12]. However, some participants even reported that they were unable to understand medication-related information due to compromised literacy and looked for additional assurance from traditional media or the Internet. To fulfill patients’ expectations of receiving clearer and individualized instructions [28], HCPs could modify information that is aligned with both the social and psychological needs related to patients’ daily lives. Future studies could conduct pre-screening on an individual’s health literacy level and elicit potential barriers to adhering to their oral targeted therapy from patients to provide tailored patient education.

A lack of two-way communication was prevalent in this study, and culturally sensitive information or interventions were needed in this patient population. While most participants generally reported obeying their physician’s orders, their perspectives on the disease and treatment often remained concealed during medical visits. Such discordant perceptions between providers and patients are widely reported and often cause decisional conflicts [29]. Some participants were not appreciative of the provider’s communication style, and expected more empathy when experiencing emotional distress and medication side-effects. Determining how to take patient’s preferences into consideration when informing them of sensitive information on cancer recurrence and changes or discontinuation of medication is a critical yet difficult task. It was also noted that half of the study participants used CAMs, specifically traditional Chinese medicine, which commonly occurs with cancer patients in East Asia [30]. Nevertheless, most participants rarely reported the use of CAMs because they were afraid that HCPs might disapprove of the concurrent use of CAMs, and this could potentially pose severe danger to patient safety due to adverse reactions [31]. Tailored interventions could thus be developed to help HCPs take the initiative in discussing these issues, especially when a patient’s preferences violated the HCP’s medical training.

Patients using targeted therapy also leveraged social support to cope with difficult tasks. In line with prior findings in patients with advanced cancer, participants perceived that the most important support was a reunion with beloved ones, and this motivated participants to strive for more time [32]. Moreover, to avoid being a burden on their family, patients with advanced cancer often hold ‘returning to normal’ as the core value in the context of living with cancer [32, 33]. However, potential concerns may occur and need to be dealt with as patients gradually take back control of their life. Although patients were familiar with managing medication-related issues, modifying and skipping prescribed doses based on their experiences may undermine the effectiveness of the medications, leading to poorer patient outcomes. In this vulnerable patient population, motivational interviewing could be integrated into the conversation to promote the positivity of taking medications as well as to keep the transparency of the drug effectiveness to reduce their concern.

Peer groups along with other supportive resources were appreciated by all participants who participated in them. Participants in this study reported positive views on receiving updated medical information and close bonding with others. It may be that patients with cancer will demand to be understood and require personalized suggestions to reduce their uncertainty [34]. However, different expectations of peer support activities were reported in patients with abundant experience. When not seeking further information, they changed their roles to support others. A possible explanation may be the lack of adequate discussion of sensitive issues, for example, of impending death and end-of-life care. While the feedback of utilizing peer support was mixed in one study [27], the benefits to emotional, informational, and social aspects were increasingly emphasized, especially in patients with advanced cancer [34]. Thus, connecting newly diagnosed patients with their peers could be a feasible intervention to fulfill mutual needs.

There were limitations to this study. First, participants were recruited from two clinics in a medical center in Taiwan; thus, findings from this research may not be applicable to different cultures or healthcare systems. Second, this study included participants who had undergone treatment with regular medical visits; thus, reasons for discontinuing oral targeted therapy or treatment failure were not well explored. However, researchers explored experiences with discontinuing medication and probed deeply into concerns from participants’ perspectives. Third, caregivers (family members or hired personnel) were allowed to accompany participants during the interviews due to the need to protect the rights of research participants. Some participants might not have felt free to discuss negative thoughts in the presence of their caregiver. Interview skills such as probing and restating the questions were used by interviewers to clarify the narratives from participants that could represent subjective experiences.

## 5. Conclusions

This study provides a deeper understanding of medication experiences, which cannot be separated from one’s daily life and shows the underlying reasoning for various medication-taking behaviors. Although values and expectations of a medication were shaped by prior experiences, patients gradually developed their own coping strategies by learning from disease-related adversities and by receiving support from HCPs and significant others. Ultimately, they changed their mindset, found appropriate ways of living with cancer, and thus returned to a ‘new’ normal. Findings from this research could help HCPs address medication-related issues by exploring patients’ holistic experiences and encouraging their ability to self-manage their disease. Interventions aimed at building connections with patients with abundant experience could be developed to empower newly diagnosed patients to better manage their disease.

## Supporting information

S1 Checklist Consolidated Criteria for Reporting Qualitative Studies (COREQ) checklist. (DOCX)

S1 Data Data matrix of themes, subthemes and codes. (DOCX)
