# Supplementary material for: Understanding the medication experience of patients with advanced non-small cell lung cancer taking epidermal growth factor receptor-tyrosine kinase inhibitors: A phenomenological study
Source: PLoS One. 2023 May 30;18(5):e0286333. doi: 10.1371/journal.pone.0286333 (PMC10228791; doi:10.1371/journal.pone.0286333)
Supplement: S1 Checklist — (DOCX) [file pone.0286333.s001.docx]

| Domain 1: Research team and reflexivity | |
| --- | --- |
| Personal characteristics | |
| Interviewer | Primary author MJW and fourth author JHW. |
| Credentials | Master student and research assistant |
| Occupation | Pharmacist and research assistant |
| Gender | Female |
| Experience & training | Training in qualitative research methods |
| Relationship with participants | |
| Relationship established prior to study commencement | No |
| Participant knowledge of the interviewer | No |
| Interviewer characteristics | No |
| Domain 2: Study design | |
| Theoretical framework | |
| Methodological orientation & theory | Phenomenology |
| Participant selection | |
| Sampling | Purposive sampling |
| Method of approach | Referred by physicians in two clinics |
| Sample size | 19 in total |
| Non-participation | Did not arise |
| Setting | |
| Setting of data collection | Private rooms in the hospital |
| Presence of non-participants | No |
| Description of sample | Outlined in Table 1 |
| Data collection | |
| Interview guide | Semi-structured guide drafted, piloted and revised |
| Repeat interviews | No repeat interviews were conducted |
| Audio/visual recording | Interviews were audio-recorded |
| Field notes | During and after interviews |
| Duration | Reported; ranged 20-60 minutes |
| Data saturation | Sampling continued until data saturation |
| Transcripts returned | No. Revised by peer debriefing |
| Domain 3: analysis and findings | |
| Data analysis | |
| Number of data coders | Two in total |
| Description of coding tree | A coding tree was developed by using thematic analysis |
| Derivation of themes | Themes were derived from the data and vivid expressions or phrases from participants |
| Software | Atlas.ti 8.0 (Scientific Software Development GmbH, Berlin, Germany) |
| Participant checking | Not conducted |
| Reporting | |
| Quotations presented | Supporting quotations presented |
| Data and findings consistent | Yes |
| Clarity of major themes | A clear presentation of major themes is outlined |
| Clarity of minor themes | Variations in views and themes and minor themes are presented. |
