# Supplementary material for: Understanding the medication experience of patients with advanced non-small cell lung cancer taking epidermal growth factor receptor-tyrosine kinase inhibitors: A phenomenological study
Source: PLoS One. 2023 May 30;18(5):e0286333. doi: 10.1371/journal.pone.0286333 (PMC10228791; doi:10.1371/journal.pone.0286333)
Supplement: S1 Data — (DOCX) [file pone.0286333.s002.docx]

| **Themes** | **Subthemes** | **Codes** |
| --- | --- | --- |
| Temporality: Values and expectations of oral targeted therapy in the terminal sentence | Unexpected death sentence from a ‘treatable’ disease | - Beliefs about cancer - Death sentence - Negative experiences from other patients - Feel lucky to be treated |
|  | Struggling to stay motivated about taking medications over the long journey | - Long-term medication - Lack of proof or objectives to manage cancer - Uncertainties about drug effectiveness |
| Corporeality: Living with adversities | Managing disease-related issues | - Refuse to accept the diagnosis - Manage medications to adapt to their schedule - Hard to digest cancer information |
|  | Distress with medication side effects | - Concern about side effects - Financial hardship |
| Relationality: Cancer diagnosis as a stepping stone or stumbling block in interpersonal relationships | Reorienting relationships | - Friends and family members can help with managing the cancer - Afraid to be a burden to family - Social disturbance |
|  | Communication and conflicts about medication issues | - Follow doctors’ instructions - Interactions with healthcare professionals - Hide the truth and feelings about their cancer - Need more tailored information |
| Spatiality: Changing lenses to bounce back to normal | Searching back and forth for an acceptable answer | - Seek medical advice - Develop their own coping strategies - Proactive monitoring |
|  | Leveraging social support to keep the faith | - Peer support and social support - Information and emotional connections |
|  | Back in the driver's seat | - Autonomy - Quality of life - Living with cancer |
